# A temporally Anchored Retrieval-Augmented Generation Framework for Metabolic and Bariatric Surgery Patient Education: An IFSO Artificial Intelligence Task Force Multinational Validation Study

**DOI:** 10.1007/s11695-026-08746-7

**Published:** 2026-05-20

**Authors:** Yash Kumar Atri, Tom Hartvigsen, Yung Lee, Allan Okrainec, Mohammad Kermansaravi, Shahab Shahabi, Silvia Leite, Mary O’Kane, Ricardo Cohen, Thomas H. Shin

**Affiliations:** 1 School of Data Science, University of Virginia, Charlottesville, USA; 2 Digestive Diseases and Surgery Institute, Cleveland Clinic, Cleveland, USA; 3 Division of General Surgery, University Health Network, Toronto, Canada; 4 Department of Surgery, Iran University of Medical Sciences, Tehran, Islamic Republic of Iran; 5 Department of Human Nutrition, University of Brasília, Brasília, Brazil; 6 Dietetic Department, Leeds Teaching Hospitals NHS Trust, Leeds, UK; 7 Center for the Treatment of Obesity and Diabetes, Hospital Alemão Oswaldo Cruz, São Paulo, Brazil; 8 Department of Surgery, University of Virginia Health System, Charlottesville, USA

**Keywords:** Retrieval-augmented generation, Large language model, Patient education, Bariatric surgery, Temporal inference, Clinical safety

## Abstract

**Background:**

Large language models (LLMs) offer promising tools for patient education, yet fixed knowledge cutoffs and hallucination risk limit their clinical utility. Current retrieval-augmented generation (RAG) approaches fail to distinguish between stable clinical knowledge and evolving recommendations.

**Methods:**

We developed and evaluated bRAGgen, a temporally anchored RAG framework incorporating five modules to enforce clinical protocols for MBS patient education: a semantic knowledge cache, multi-source evidence retrieval with graph-based fusion, uncertainty-aware generation, clinical constraint reranking, and Temporal Fisher Anchoring with Mechanism Selectivity (TFAMS) for adaptive inference. The framework was evaluated using 105 expert-curated free-response questions assessed by a multinational panel of seven specialists (5 surgeons, 2 dietitians) from five countries on a 5-point Likert scale for factuality, clinical relevance, and comprehensiveness. LLM-as-Judge evaluation using ChatGPT-4o provided complementary automated assessment.

**Results:**

bRAGgen significantly improved response quality across all five base language models tested (*p* < 0.001), with large effect sizes for higher-capacity models (Cohen’s d = 0.96–1.01) and moderate effects for smaller models (Cohen’s d = 0.38–0.56) with good inter-rater reliability (Krippendorff’s α = 0.72). The largest gains occurred in safety-critical categories including Risks and Complications (+ 1.84 points) and Mental and Emotional Health (+ 1.84 points), suggesting the framework is most impactful where nuanced clinical judgment is essential. LLM-as-Judge evaluation using ChatGPT-4o demonstrated high concordance with expert ratings (Spearman’s ρ = 0.94).

**Conclusions:**

This proof-of-concept study suggests that a multi-module RAG framework with temporal stability anchoring can improve expert-rated LLM response quality for bariatric surgery domain knowledge, though prospective validation in patient-facing settings is needed before clinical implementation.

**Supplementary Information:**

The online version contains supplementary material available at 10.1007/s11695-026-08746-7.

## Key Points

- bRAGgen improved expert-rated LLM response quality across all models tested.
- Largest gains in safety-critical domains: complications and mental health.
- Multinational panel of seven MBS experts from five countries validated results.
- LLM-as-Judge showed high concordance with expert ratings (ρ = 0.94).

**Supplementary Information:**

The online version contains supplementary material available at 10.1007/s11695-026-08746-7.

## Introduction

Metabolic and bariatric surgery (MBS) remains the gold standard treatment for severe obesity and metabolic disease, with over 270,000 annual procedures in the United States [1–5]. However, successful weight loss post-MBS relies in part on patient education surrounding dietary and lifestyle modifications, postoperative complications, and psychosocial support, emphasizing the need for optimal education strategies for patients preparing for MBS [2, 6–10].

Overall, the lack of sustained patient engagement and education post-MBS is a critical impediment to optimal postoperative outcomes with many causes, including low health literacy rates, information inaccessibility, and geographic distances to healthcare providers [2, 5, 11, 12].Given these challenges, there is a need for scalable, accessible, and continually updated educational and decision support tools tailored to the unique needs of MBS patients with contemporary data [13]. Traditional patient education materials, whether delivered in print, via static websites, or through periodic telehealth visits, often fail to adapt dynamically to emerging clinical evidence or evolving clinical status of individual patients [14]. Moreover, existing digital health platforms seldom incorporate mechanisms to detect when their guidance may be outdated or insufficiently confident, leading to knowledge gaps in both patients and clinicians [15, 16].

Large language models (LLMs) offer a potential solution by providing natural language interfaces for patients to query [17–19]. However, LLMs face limitations due to fixed knowledge cutoffs and the most-capable are trained on broad, general-purpose corpora, leaving them unaware of the latest bariatric surgery guidelines or nuanced postoperative considerations [20–23]. To address this, we propose bRAGgen, a temporally anchored inference framework grounded in real-world clinical dynamics that leverages Temporal Fisher Anchoring with Mechanism Selectivity (TFAMS). The model is tested against a corpus of 1,302 MBS domain-specific free-response questions derived from biomedical literature and expert-informed synthetic generation, from which a curated subset of 105 questions was validated by board-certified bariatric surgeons for expert evaluation [24, 25]. In a two-phase evaluation, leveraging both LLM-based metrics and expert surgeon reviews, bRAGgen demonstrates improved performance in generating clinically accurate, relevant, and temporally coherent responses, suggesting potential for more reliable, evidence-based support for MBS patients in an evolving medical landscape.

## Methods

### Development of a Temporal Anchored Inference Framework for Enhanced Retrieval-Augmented Generation

**Fig. 1:**
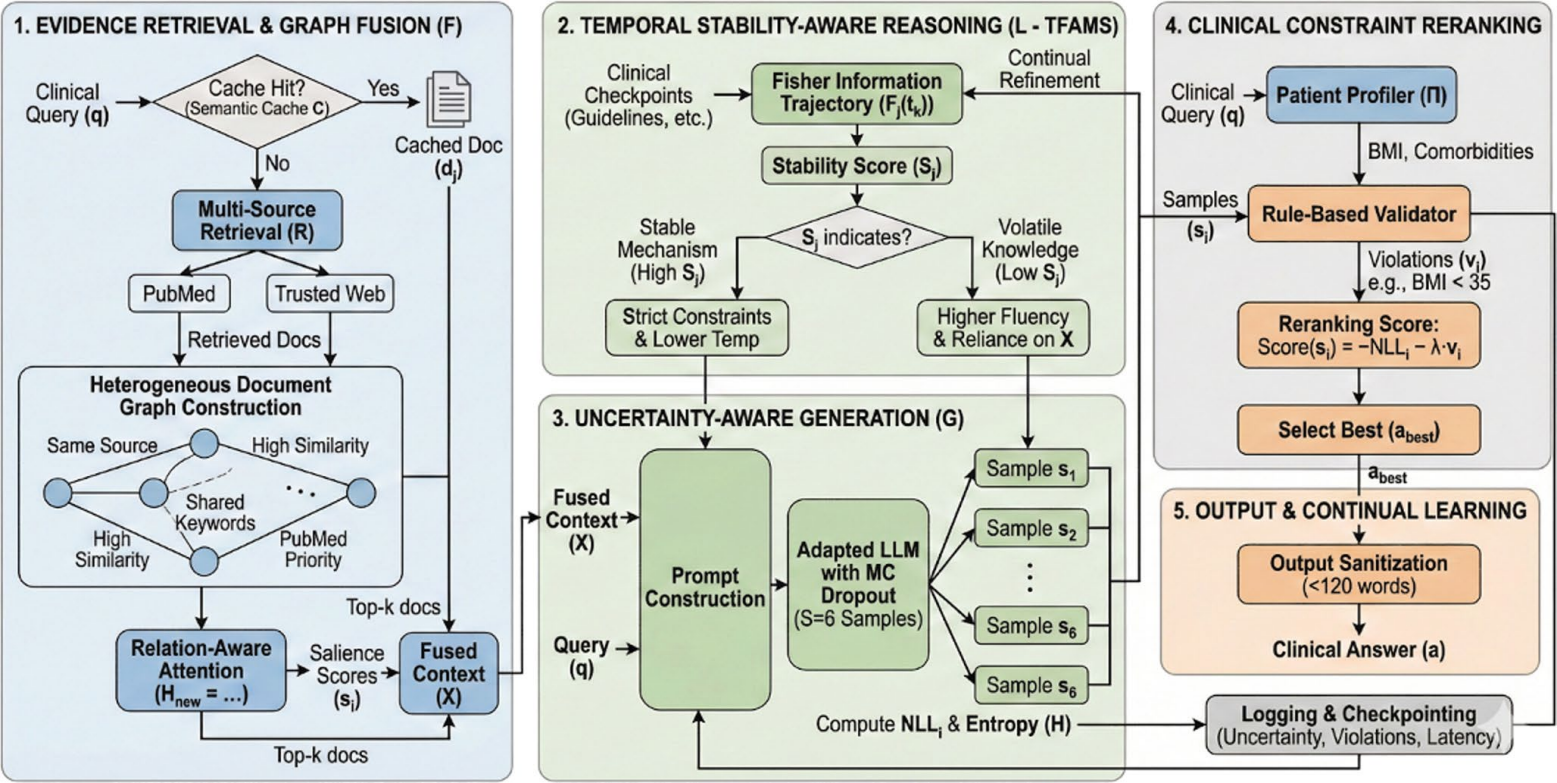
Architecture of the proposed bariatric surgery domain-specific LLM framework bRAGgen. The system integrates LLM with real-time web retrieval capabilities. When confidence falls below the threshold, the system automatically retrieves updated information from authoritative medical sources to enhance response accuracy

To integrate temporal awareness and adaptability into an LLM framework, we developed bRAGgen, an enhanced retrieval-augmented generation (RAG) LLM model that leverages TFAMS, enabling multi-source evidence fusion, uncertainty-aware generation, clinical constraint enforcement, and temporal stability-aware inference (Fig. 1). TFAMS constructs time-indexed Fisher information trajectories from trusted clinical interactions and historical checkpoints (e.g., guideline releases) to identify model behaviors associated with temporally stable mechanisms. These stable directions are protected during inference, while adaptation is selectively permitted only along historically volatile knowledge subspaces. When uncertainty is detected, bRAGgen retrieves the latest evidence from peer-reviewed sources on PubMed while ensuring corpus integration respects the boundary between evolving recommendations and invariant clinical truths.

### Generation of MBS Domain-Specific LLM bRAGgen Framework and Dataset

The framework comprises five integrated modules: (1) a semantic knowledge cache that stores previously validated clinical evidence using vector embeddings for rapid sub-second retrieval; (2) a multi-source evidence retrieval engine that queries PubMed and curated medical web sources when cached evidence is insufficient, synthesizing heterogeneous results through a graph-based fusion mechanism that prioritizes peer-reviewed literature; (3) an uncertainty-aware text generation module that uses Monte Carlo dropout to quantify predictive confidence, deferring to retrieval when confidence is insufficient for safe clinical guidance; (4) a clinical constraint reranking system that validates generated responses against bariatric surgery protocols, flagging violations such as inappropriate eligibility claims, contraindicated medication recommendations, or omission of required follow-up monitoring; and (5) TFAMS, which uses the Fisher Information Matrix as a temporal sensor of knowledge stability—anchoring responses to invariant clinical truths (e.g., NSAID contraindications post-operatively) while permitting adaptation along historically volatile knowledge subspaces (e.g., evolving BMI eligibility thresholds). Detailed mathematical formulations for each module are provided in Supplementary Methods.

To support development of intelligent tools for MBS patient education and clinical decision support, a domain-specific free-response question (FRQ) dataset comprising 1,302 questions was curated from two primary sources: 611 questions derived from PubMedQA, of which 201 were flagged by expert reviewers as not representative of everyday patient concerns but retained in the corpus, and 691 questions synthetically generated using expert-informed templates and real-world patient interactions. From this set, 105 questions were selected through expert collaboration and consensus by board-certified bariatric surgeons to ensure clinical validity and relevance with a benchmark designed to evaluate clinical knowledge accuracy and comprehensiveness as a prerequisite for patient education applications, rather than to assess patient-facing communication style directly. Questions spanned over a wide range of themes, including preoperative considerations, intraoperative topics, postoperative management, dietary guidance, mental health, and lifestyle adaptation. This 105-question subset served as the primary evaluation set for expert review and statistical comparisons. The broader 1,302-question corpus was used for model training and exploratory benchmarking.

### Experimental Setup

The LLM framework and models were evaluated using our curated 105-question FRQ dataset. A multidisciplinary expert panel of seven providers (5 bariatric surgeons, 2 bariatric dietitians) representing practices from 5 countries (United States, Canada, United Kingdom, Iran, Brazil) across academic and private settings independently rated all responses. Each panelist rated every question across all model conditions. Raters were blinded to model identity and experimental condition. The panel used a 5-point Likert scale with predefined anchors (1 = factually incorrect or potentially harmful; 5 = fully accurate, comprehensive, and clinically appropriate) to assess responses for factuality (accuracy and correctness), clinical relevance (context appropriateness), and comprehensiveness (completeness). Scores were aggregated as mean ratings per question per condition. Two panelists contributed to initial question curation but did not participate in selecting the final 105-question evaluation subset and were blinded to model outputs during rating. To complement expert review and enable scalable comparison, responses were also evaluated using ChatGPT-4o in an LLM-as-Judge framework applying the same 5-point Likert scale.

### Statistical Analyses

Model performance is summarized in Results tables as mean scores on 5-point Likert scale unless otherwise specified. Paired Wilcoxon signed-rank tests with Benjamini-Hochberg FDR correction were used to assess statistical significance in model improvement with bRAGgen based on the 105-question expert-curated evaluation set. Meaningful effect size was determined using Cohen’s d. Inter-rater agreement was assessed using Krippendorff’s α with an ordinal distance metric over 5-point Likert scale. Spearman rank correlation was calculated to assess inter-expert and expert-ChatGPT4o agreement across models for each domain. As a sensitivity analysis to account for the repeated and clustered structure of the data, a linear mixed-effects model was fit with Likert score as the outcome, system configuration as a fixed effect, and question ID and rater ID as random intercepts. The use of linear models for 5-point Likert-scale data is supported by evidence that parametric approaches are robust to ordinal scaling at this measurement level [26].

## Results

### Dataset Curation and Baseline Model Performance

A composite dataset of 1,302 MBS domain-specific FRQs was curated from PubMedQA-derived questions (*n* = 611) and synthetically generated queries based on expert-informed templates and patient interactions (*n* = 691) [27]. Of the PubMedQA-derived questions, 201 were flagged by experts as less representative of typical patient concerns but were retained for training purposes. The corpus spans a variety of MBS-related themes, including Recovery and Lifestyle (22.7%), Mental and Emotional Health (22.0%), Surgical and Medical information (20.0%), Risks and Complications (17.0%), Nutrition and Diet (7.8%), Preparation and Logistics (5.2%), and Cost and Insurance (5.0%; Supplemental Table 1). Offline RAG^2^ and MedGraphRAG were employed to establish baseline performance using standard RAG and domain-tuned graph-based retrieval, yielding mean scores of 3.38 and 3.96, respectively (Table 1) [28, 29]. Representative questions from each category are provided in Supplemental Table 2. Expert panel evaluation was conducted on the 105-question curated subset, representing a clinically grounded evaluation set while the broader corpus was used for exploratory benchmarking.

**Table 1:** bRAGgen performance of bariatric surgery domain-specific questions evaluated by expert panel

System	Metrics (mean Likert score)			
	Factuality	Clinical Relevance	Comprehensiveness	Overall
Zero-shot				
Hermes-Llama3-8B	2.34	2.11	2.59	2.35
Qwen2.5-7B-Chat	2.25	2.06	2.41	2.15
BioMistral-7B	2.63	2.45	2.92	2.63
Meditron-7B	2.74	2.55	2.83	2.68
Phi-3-mini-12-8k	2.08	1.93	2.21	2.08
Standard RAG				
RAG^2^	3.49	3.28	3.36	3.38
MedGraphRAG	3.67	3.76	4.45	3.96
Context-prompted				
Hermes-Llama3-8B	3.67	3.76	4.45	3.96
Qwen2.5-7B-Chat	3.62	3.7	4.38	3.9
BioMistral-7B	2.57	2.24	3.21	2.67
Meditron-7B	2.5	2.2	2.3	2.33
Phi-3-mini-12-8k	2.49	2.61	2.38	2.49
Fine-Tuned				
Hermes-Llama3-8B	3.61	3.68	4.32	3.87
Qwen2.5-7B-Chat	3.55	3.62	4.25	3.81
BioMistral-7B	2.48	2.15	3.1	2.58
Meditron-7B	2.42	2.1	2.22	2.25
Phi-3-mini-12-8k	2.4	2.52	2.3	2.41
bRAGgen				
Hermes-Llama3-8B	4.03	4.43	4.87	4.44
Qwen2.5-7B-Chat	3.98	4.35	4.78	4.37
BioMistral-7B	3.55	3.62	3.52	3.56
Meditron-7B	3.48	3.55	3.42	3.48
Phi-3-mini-12-8k	2.73	3.03	2.54	2.77

Scores based on 5-point Likert Scale, 1 = lowest performance to 5 = highest performance

### Adaptive RAG Model bRAGgen Significantly Improves Expert-Rated LLM Response Quality

We additionally evaluated the following models to assess zero-shot and context-prompted LLMs performance: Llama3-8B, Qwen1.5, Phi-3, Meditron, and Mistral Instruct [17, 18, 30]. Mean scores on blinded expert-evaluated Likert scale in the zero-shot setting were lowest across all models (average scores: Llama3-8b 2.35, Qwen2.5-7b 2.15, BioMistral-7b 2.63, Meditron-7b 2.68, and Phi-3 2.08). Performance scores were highest in context-prompted Llama3-8B where mean factuality, clinical relevance, and comprehensiveness were 3.67, 3.76, and 4.45, respectively (Table 1). Context-prompt configuration improved all models’ performances compared to fine-tuned by an average of 0.09 points. However, these scores significantly improved for all models with implementation of our adaptive bRAGgen framework, with Llama3-8B remaining the best performing LLM based on mean factuality, clinical relevance, and comprehensiveness scores of 4.03, 4.43, and 4.87. bRAGgen demonstrated a 0.48 mean score improvement compared to context-prompted Llama-3.8B (Factuality + 0.36, Clinical Relevance + 0.67, Comprehensiveness + 0.42) and an overall mean score improvement of 0.65 across all models. Performance improvement by bRAGgen achieved statistical significance across all base models and comparison conditions based on paired Wilcoxon signed-rank tests with Benjamini-Hochberg correction (Table 2). Higher-capacity models had the largest improvement effect sizes over zero-shot (Llama3-8B + 1.19 points, *p* < 0.001, Cohen’s d = 1.01; Qwen2.5-7B + 1.14 points, *p* < 0.001, Cohen’s d = 0.96; BioMistral-7B + 0.98 points, *p* < 0.001, Cohen’s d = 0.84). Though with more modest effect sizes (Cohen’s d range 0.38–0.56), bRAGgen was able to produce similar improvements with smaller language models such as Phi-3-mini. Inter-rater reliability among the seven expert panelists was acceptable with Krippendorff’s α of 0.72 overall. By domain, there was good inter-rater reliability for Factuality (Krippendorff’s α = 0.68) and Clinical Relevance (α = 0.71), with moderate agreement for Comprehensiveness (α = 0.63). These findings were confirmed by a linear mixed-effects model accounting for clustering by rater and question. Relative to the zero-shot baseline, bRAGgen demonstrated the largest improvement with an estimated increase of + 2.19 Likert points (95%CI [2.09, 2.29], *p* < 0.001; Supplemental Table 3).

**Table 2:** Paired comparisons of model performance between bRAGgen and baseline configurations

Base model	Comparison	ΔScore	*p*	Cohen’s d
Hermes-Llama3-8B	bRAGgen vs. Zero-shot	+ 1.19	< 0.001	1.01
	vs. Context-prompted	+ 0.47	< 0.001	0.58
	vs. Fine-tuned	+ 0.56	< 0.001	0.69
Qwen2.5-7B-Chat	bRAGgen vs. Zero-shot	+ 1.14	< 0.001	0.96
	vs. Context-prompted	+ 0.44	< 0.001	0.54
	vs. Fine-tuned	+ 0.52	< 0.001	0.63
BioMistral-7B	bRAGgen vs. Zero-shot	+ 0.98	< 0.001	0.84
	vs. Context-prompted	+ 0.38	0.001	0.47
	vs. Fine-tuned	+ 0.45	< 0.001	0.55
Meditron-7B	bRAGgen vs. Zero-shot	+ 0.91	< 0.001	0.78
	vs. Context-prompted	+ 0.35	0.002	0.44
	vs. Fine-tuned	+ 0.41	0.001	0.51
Phi-3-mini-12-8k	bRAGgen vs. Zero-shot	+ 0.66	0.001	0.56
	vs. Context-prompted	+ 0.29	0.004	0.38
	vs. Fine-tuned	+ 0.33	0.003	0.42

ΔScore represents change in median score from 5-point Likert scale, 1 = lowest performance to 5 = highest performance; Statistical significance assessed by paired Wilcoxon signed-rank tests with Benjamini-Hochberg FDR correction and Cohen’s d to report effect size

### Category-Specific Performance Analysis of bRAGgen with Llama3-8B

To determine bRAGgen effectiveness across clinical domains, we analyzed expert evaluation scores stratified by question category (Supplemental Table 4). Overall, bRAGgen augmented Llama3-8B performance across all domains. The largest gains were evidence in Risks and Complications (+ 1.84 points) and Mental and Emotional Health (+ 1.84 points). These were followed by Recovery and Lifestyle (+ 1.66 points) and Surgical/Medical Information (+ 1.37 points). The smallest improvements were seen in Preparation and Logistics (+ 1.12 points) and Cost and Insurance (+ 1.10 points) categories. suggesting that bRAGgen’s framework mechanisms confer the greatest benefit for queries related to nuanced clinical judgement and up-to-date evidence rather than logistical or administrative content where static knowledge may be sufficient.

### Concordance of LLM-as-Judge Evaluation with Expert Consensus Assessment of bRAGgen

To complement expert evaluation, we further assessed all models using an LLM-as-Judge framework, where we used ChatGPT-4o model scores responses along Factuality, Clinical Relevance, and Comprehensiveness using a 5-point Likert scale (Table 3). These results were compared against scores by category and model derived from expert panel consensus evaluation. Overall, there is a high degree of consistency in relative rankings across systems. Both experts and ChatGPT4o identify MedGraphRAG and context-prompted Llama3-8B as the strongest baselines, while zero-shot models like Phi-3 and Mistral perform the worst across all axes. Furthermore, addition of bRAGgen framework yields the highest scores in both evaluation schemes, affirming its robustness across human and model-based judgments. Importantly, the average correlation between expert and LLM-as-Judge scores across all models is Spearman’s ρ = 0.94. Metric-wise, the strongest agreement is seen in the Comprehensiveness and Clinical Relevance dimensions, where score trends closely track each other across settings.

**Table 3:** bRAGgen performance of bariatric surgery domain-specific questions evaluated by LLM-as-Judge

System	Metrics (mean Likert score)			
	Factuality	Clinical Relevance	Comprehensiveness	Overall
Zero-shot				
Hermes-Llama3-8B	2.39	2.18	2.57	2.36
Qwen2.5-7B-Chat	2.29	2.12	2.48	2.32
BioMistral-7B	2.87	2.73	3.04	2.88
Meditron-7B	2.92	2.81	3.12	2.95
Phi-3-mini-12-8k	2.14	1.97	2.26	2.12
Standard RAG				
RAG^2^	2.91	2.76	3.05	2.91
MedGraphRAG	3.48	3.62	3.95	3.68
Context-prompted				
Hermes-Llama3-8B	3.82	3.91	4.29	4.01
Qwen2.5-7B-Chat	3.65	3.83	4.12	3.87
BioMistral-7B	2.94	2.67	3.1	2.9
Meditron-7B	2.21	2.55	2.74	2.5
Phi-3-mini-12-8k	2.37	2.12	2.66	2.38
Fine-Tuned				
Hermes-Llama3-8B	3.56	3.64	3.98	3.73
Qwen2.5-7B-Chat	3.42	3.51	3.87	3.6
BioMistral-7B	3.1	2.78	3.32	3.07
Meditron-7B	2.45	2.69	2.81	2.65
Phi-3-mini-12-8k	2.18	2.42	2.25	2.28
bRAGgen				
Hermes-Llama3-8B	4.25	4.52	4.89	4.55
Qwen2.5-7B-Chat	4.18	4.47	4.82	4.49
BioMistral-7B	3.72	3.85	3.94	3.84
Meditron-7B	3.61	3.77	3.88	3.75
Phi-3-mini-12-8k	3.05	3.22	3.18	3.15

Scores based on 5-point Likert Scale, 1 = lowest performance to 5 = highest performance

### System Calibration and Inference Dynamics of bRAGgen Framework

**Fig. 2:**
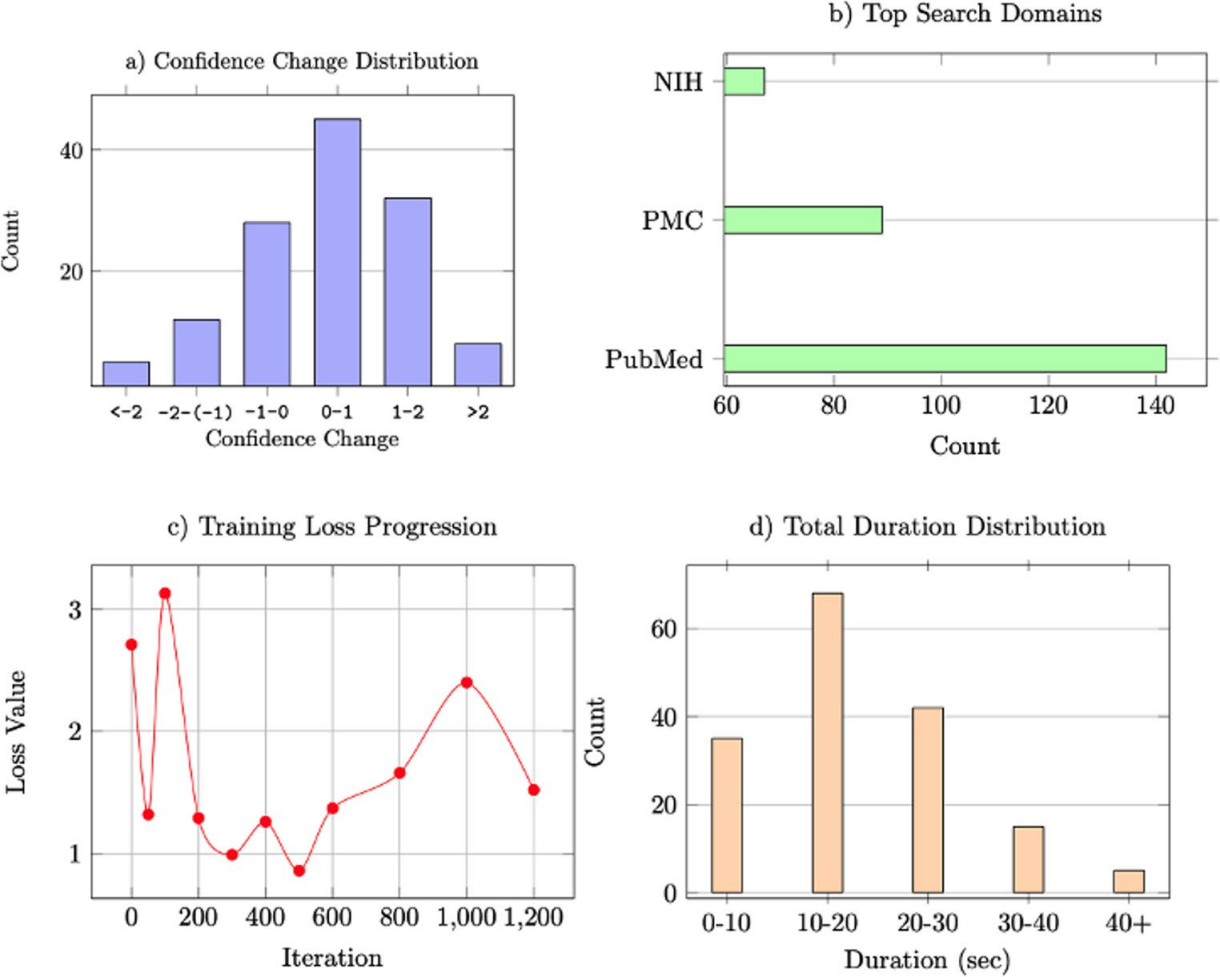
Exploratory analyses of system calibration and inference dynamics: (**a**) distribution of changes in confidence scores post-edit, showing that most changes are modest and positive; (**b**) frequency of search queries across external biomedical domains with PubMed predominance; (**c**) loss trajectory during offline LoRA domain adaptation, illustrating convergence of the one-time fine-tuning process prior to deployment; (**d**) distribution of total duration taken for each edit operation, highlighting that most edits are executed within 10–20 s

We performed analyses to characterize bRAGgen’s inference-time behavior and offline calibration. At inference time, the distribution of confidence score changes (Fig. 2A) showed that bRAGgen’s dynamic thresholding yields frequent yet stable adjustments without overcorrecting. PubMed, PMC, and NIH emerged as the top knowledge sources during retrieval (Fig. 2B), underscoring alignment with authoritative biomedical repositories. The loss trajectory across 1,302 training iterations during LoRA domain adaptation (Fig. 2C) demonstrated initial rapid improvement followed by gradual convergence, confirming stable offline fine-tuning prior to deployment. This domain adaptation is performed once and is distinct from the TFAMS inference mechanism, which does not modify model parameters. Most inference operations completed within 10–20 s (Fig. 2D), validating computational feasibility for interactive clinical settings. A pseudocode representation of the complete inference pipeline, including TFAMS stability modulation, is provided in Supplemental Materials.

### Component-Wise Ablation Analysis

To evaluate the incremental contribution of each module, we performed a progressive ablation study using Hermes-Llama3-8B as the base model under the LLM-as-Judge evaluation protocol, given its high concordance with expert ratings (Spearman’s ρ = 0.94). Starting from the zero-shot base model, we sequentially added retrieval, graph-based evidence fusion, semantic caching, clinical constraint reranking, and TFAMS (Table 4). Each component contributed to progressive improvement in factuality, clinical relevance, and comprehensiveness. Retrieval and graph-based fusion provided the largest gains, while TFAMS contributed an additional + 0.19-point improvement over the full pipeline without temporal anchoring, representing the second-largest individual module contribution. Monte Carlo dropout-based uncertainty estimation is integral to the stochastic decoding process and cannot be meaningfully isolated as a standalone module; and therefore incorporated within the generation stage throughout all ablation conditions.

**Table 4:** Ablation analysis showing incremental contribution of each component in bRAGgen framework using Hermes-Llama3-8B as the base model under the LLM-as-Judge evaluation protocol

System Variant	Factuality	Clinical Relevance	Comprehensiveness	Overall
Base Model (Hermes-Llama3-8B, zero-shot)	2.39	2.18	2.57	2.36
+ Semantic caching	2.61	2.46	2.83	2.63
+ Retrieval (standard RAG)	3.82	3.91	4.29	4.01
+ Graph-based evidence fusion	4.08	4.28	4.61	4.32
+ Clinical constraint re-ranking	4.10	4.31	4.66	4.36
+ TFAMS	4.25	4.52	4.89	4.55

Scores based on 5-point Likert Scale, 1 = lowest performance to 5 = highest performance

## Discussion

In this study, we present a proof-of-concept illustrating performance augmentation of retrieval-augmented generation for LLM-based MBS patient education by incorporating TFAMS, enabling an adaptive and temporally anchored framework for continued learning within a clinical domain. Based on review by a multidisciplinary panel of bariatric surgery experts, the proposed bRAGgen framework delivered significantly improved scores across all metrics. When paired with Llama3-8B, bRAGgen achieved the highest average score overall (4.51), with particularly strong performance in Comprehensiveness and Clinical Relevance. bRAGgen improved expert-rated factuality, suggesting potential for reduced clinically relevant inaccuracies, while remaining adaptive to new data without sacrificing performance on established clinical knowledge.

These gains highlight the effectiveness of our confidence-aware updating mechanism, which retrieves up-to-date clinical evidence and integrates it into the model’s reasoning, enabling more robust domain-adapted responses [31–35]. bRAGgen significantly enhances the clinical utility of LLMs across model sizes compared to conventional static RAG setups or prompting-only strategies, supported by statistical significance on paired Wilcoxon signed-rank testing with moderate-to-large effect sizes [36–39]. Notably, bRAGgen’s uncertainty-aware retrieval and constraint-guided generation preferentially augmented performance in knowledge areas dependent on updated evidence and nuanced clinical decision-making over questions related to more static content.

Even with smaller models like Phi-3 and Mistral Instruct, bRAGgen enhances output quality, particularly in relevance and completeness, making it practical for resource-constrained settings. High concordance between ChatGPT-4o and the expert panel (Spearman’s ρ = 0.94) underscores the potential utility of LLMs as surrogate evaluators in iterative development settings. However, minor variation in Factuality scores for smaller models may reflect the LLM’s heightened sensitivity to surface-level inaccuracies compared to domain experts who weigh overall clinical soundness more heavily [37, 38]. LLM-as-judge evaluation reflects consistency in relative model rankings, rather than independent validation of clinical correctness.

While bRAGgen demonstrates strong performance, several limitations remain as open avenues for further research. Although the method enables localized and compositional edits, the cumulative impact of many such edits – particularly in dense regions of the representation space – may lead to interference or capacity saturation. Future work could explore dynamic pruning or hierarchical edit graphs to support long-term scalability. While effective at modifying behaviors related to specific factual updates, bRAGgen’s ability to generalize edits to broader semantic or reasoning contexts is limited. Integrating structured world knowledge or training the modules with auxiliary objectives (e.g., counterfactual consistency) may further enhance generalization. While our automatic and human evaluations provide evidence of improved edit quality, assessing real-world applicability in high-stakes domains requires more robust metrics. Developing editing-specific benchmarks that reflect user intent, edit trustworthiness, and long-term retention would be a valuable direction. Further work is needed to refine confidence thresholds to better filter unreliable inputs and variable data quality. The current evaluation benchmark primarily assesses clinical knowledge accuracy rather than patient-facing communication quality. While clinically accurate outputs are a necessary foundation for patient education tools, the benchmark does not evaluate whether responses are comprehensible to lay audiences or phrased in patient-appropriate language. Future work should incorporate patient-facing evaluation using lay-language questions, readability assessment, and patient comprehension outcomes to validate the framework’s suitability for direct patient interaction. Translation to real-world patient use will require prospective validation including usability testing, assessment of end-user comprehension, and clinical outcomes. The framework’s modular architecture may be adaptable to other clinical specialties with reconfiguration of domain-specific constraints and retrieval sources. The current evaluation relies on a static benchmark and does not include a time-sensitive comparison (e.g., pre- versus post-guideline update). While the ablation analysis demonstrates that TFAMS contributes incremental gains beyond retrieval and constraint mechanisms, prospective evaluation using temporally dynamic benchmarks is needed to fully characterize the temporal anchoring mechanism.

## Conclusion

This proof-of-concept study demonstrates that a multi-module RAG framework incorporating temporal stability anchoring, evidence fusion, and clinical constraint enforcement can improve expert-rated LLM response quality for bariatric surgery domain knowledge. These findings do not establish readiness for patient-facing deployment or clinical implementation. Translation to real-world patient education will require prospective validation including usability testing with patient populations, assessment of lay comprehension, and evaluation of clinical outcomes. The framework’s modular architecture may be adaptable to other clinical specialties with reconfiguration of domain-specific constraints and retrieval sources.

## Electronic Supplementary Material

Below is the link to the electronic supplementary material.

Supplementary Material 1

## Data Availability

No datasets were generated or analysed during the current study.

## Code Availability

The materials necessary to reproduce the statistical analyses and LLM framework will be openly available upon publication in a public repository, to include code for data processing, statistical testing, prompt templates, model configuration, and RAG pipeline. No patient data were used in this study.

## References

[1] Clapp B, Ponce J, Corbett J, Ghanem OM, Kurian M, Rogers AM, et al. American Society for Metabolic and Bariatric Surgery 2022 estimate of metabolic and bariatric procedures performed in the United States. Surg Obes Relat Dis. 2024;20:425–31. 10.1016/j.soard.2024.01.012. 38448343 10.1016/j.soard.2024.01.012

[2] Mechanick JI, Apovian C, Brethauer S, Timothy Garvey W, Joffe AM, Kim J et al. Clinical Practice Guidelines for the Perioperative Nutrition, Metabolic, and Nonsurgical Support of Patients Undergoing Bariatric Procedures – 2019 Update: Cosponsored by American Association of Clinical Endocrinologists/American College of Endocrinology, The Obesity Society, American Society for Metabolic and Bariatric Surgery, Obesity Medicine Association, and American Society of Anesthesiologists. Obesity (Silver Spring). 2020;28:O1–58. 10.1002/oby.22719 10.1002/oby.22719 32202076

[3] Barres R, Kirchner H, Rasmussen M, Yan J, Kantor FR, Krook A, et al. Weight loss after gastric bypass surgery in human obesity remodels promoter methylation. Cell Rep. 2013;3:1020–7. 10.1016/j.celrep.2013.03.018. 23583180 10.1016/j.celrep.2013.03.018

[4] Loos RJF, Yeo GSH. The genetics of obesity: from discovery to biology. Nat Rev Genet. 2022;23:120–33. 10.1038/s41576-021-00414-z. 34556834 10.1038/s41576-021-00414-z PMC8459824

[5] Setarehdan SA, Sheidaei A, Mokhber S, Varse F, Pazouki A, Solaymani-Dodaran M. Determinants of Patient’s Adherence to the Predefined Follow-up Visits After Bariatric Surgery. Obes Surg. 2023;33:577–84. 10.1007/s11695-022-06428-8. 36572837 10.1007/s11695-022-06428-8 PMC9792310

[6] Groller KD. Systematic review of patient education practices in weight loss surgery. Surg Obes Relat Dis. 2017;13:1072–85. 10.1016/j.soard.2017.01.008. 28216118 10.1016/j.soard.2017.01.008

[7] Bjerkan KK, Sandvik J, Nymo S, Græslie H, Johnsen G, Mårvik R, et al. The Long-Term Impact of Postoperative Educational Programs on Weight Loss After Roux-en-Y Gastric Bypass. Obes Surg. 2022;32:3005–12. 10.1007/s11695-022-06187-6. 35790673 10.1007/s11695-022-06187-6 PMC9392699

[8] David LA, Sijercic I, Cassin SE. Preoperative and post-operative psychosocial interventions for bariatric surgery patients: A systematic review. Obes Rev. 2020;21:e12926. 10.1111/obr.12926. 31970925 10.1111/obr.12926

[9] McLennan S, Verhoeff K, Mocanu V, Jogiat U, Birch DW, Karmali S, et al. Characteristics and outcomes for patients undergoing revisional bariatric surgery due to persistent obesity: a retrospective cohort study of 10,589 patients. Surg Endosc. 2023;37:4613–22. 10.1007/s00464-023-09951-6. 36859722 10.1007/s00464-023-09951-6

[10] Kim DH, Lukens FJ, Ko D, Salazar M, Kröner PT, Elli EF, et al. Incidence, Burden, and Predictors of 11-Month Readmission in Patients Undergoing Bariatric Surgery. Obes Surg. 2023;33:94–104. 10.1007/s11695-022-06343-y. 36319825 10.1007/s11695-022-06343-y

[11] Schlottmann F, Baz C, Pirzada A, Masrur MA. Postoperative Follow-up Compliance: The Achilles’ Heel of Bariatric Surgery. Obes Surg. 2023;33:2945–8. 10.1007/s11695-023-06769-y. 37505342 10.1007/s11695-023-06769-y

[12] Bartholomay EM, Cox S, Tabone L, Szoka N, Abunnaja S, Aylward L. Sociodemographic factors related to bariatric follow-up appointment attendance and weight outcomes. Surg Obes Relat Dis. 2024;20:1388–95. 10.1016/j.soard.2024.08.010. 39256114 10.1016/j.soard.2024.08.010

[13] G PJ, Chopra GVVK, Emran H. Enhancing postoperative care with telemedicine and remote monitoring for improved recovery and patient safety. Int J Surg. 2024;110:8205–6. 10.1097/JS9.0000000000002132. 39504348 10.1097/JS9.0000000000002132 PMC11634097

[14] Javanparast S, Roeger L, Kwok Y, Reed RL. The experience of Australian general practice patients at high risk of poor health outcomes with telehealth during the COVID-19 pandemic: a qualitative study. BMC Fam Pract. 2021;22:69. 10.1186/s12875-021-01408-w. 33832422 10.1186/s12875-021-01408-w PMC8031338

[15] Wang X, Zhang NX, He H, Nguyen T, Yu K-H, Deng H et al. Safety challenges of AI in medicine in the era of large language models [Internet]. arXiv; 2025 [cited 2026 Jan 13]. 10.48550/arXiv.2409.18968

[16] Narayan SM, Kohli N, Martin MM. Addressing contemporary threats in anonymised healthcare data using privacy engineering. NPJ Digit Med. 2025;8:145. 10.1038/s41746-025-01520-6. 40050672 10.1038/s41746-025-01520-6 PMC11885643

[17] Grattafiori A, Dubey A, Jauhri A, Pandey A, Kadian A, Al-Dahle A et al. The Llama 3 Herd of Models [Internet]. arXiv; 2024 [cited 2026 Jan 13]. 10.48550/arXiv.2407.21783.

[18] Abdin M, Aneja J, Awadalla H, Awadallah A, Awan AA, Bach N et al. Phi-3 Technical Report: A Highly Capable Language Model Locally on Your Phone [Internet]. arXiv; 2024 [cited 2026 Jan 13]. 10.48550/arXiv.2404.14219

[19] Minaee S, Mikolov T, Nikzad N, Chenaghlu M, Socher R, Amatriain X et al. Large Language Models: A Survey [Internet]. arXiv; 2025 [cited 2026 Jan 13]. 10.48550/arXiv.2402.06196

[20] Cheng J, Marone M, Weller O, Lawrie D, Khashabi D, Durme BV. Dated Data: Tracing Knowledge Cutoffs in Large Language Models [Internet]. arXiv; 2024 [cited 2026 Jan 13]. 10.48550/arXiv.2403.12958

[21] Alber DA, Yang Z, Alyakin A, Yang E, Rai S, Valliani AA, et al. Medical large language models are vulnerable to data-poisoning attacks. Nat Med. 2025;31:618–26. 10.1038/s41591-024-03445-1. 39779928 10.1038/s41591-024-03445-1 PMC11835729

[22] Bélisle-Pipon J-C. Why we need to be careful with LLMs in medicine. Front Med (Lausanne). 2024;11:1495582. 10.3389/fmed.2024.1495582. 39697212 10.3389/fmed.2024.1495582 PMC11652181

[23] Lee Y, Shin T, Tessier L, Javidan A, Jung J, Hong D, et al. Harnessing artificial intelligence in bariatric surgery: comparative analysis of ChatGPT-4, Bing, and Bard in generating clinician-level bariatric surgery recommendations. Surg Obes Relat Dis. 2024;20:603–8. 10.1016/j.soard.2024.03.011. 38644078 10.1016/j.soard.2024.03.011

[24] Nguyen NT, Hutter MM, Ikramuddin S, Maria EJ, editors. The SAGES manual: a practical guide to bariatric surgery. New York, NY: Springer New York; 2008. 10.1007/978-0-387-69171-8.

[25] Nguyen NT. In: Blackstone RP, Morton JM, Ponce J, Rosenthal RJ, editors. The ASMBS Textbook of Bariatric Surgery: Volume 1: Bariatric Surgery. New York, NY: Springer; 2015. 10.1007/978-1-4939-1206-3.

[26] Norman G. Likert scales, levels of measurement and the laws of statistics. Adv Health Sci Educ Theory Pract. 2010;15:625–32. 10.1007/s10459-010-9222-y. 20146096 10.1007/s10459-010-9222-y

[27] Jin Q, Dhingra B, Liu Z, Cohen W, Lu X, PubMedQA:. A Dataset for Biomedical Research Question Answering. Proceedings of the 2019 Conference on Empirical Methods in Natural Language Processing and the 9th International Joint Conference on Natural Language Processing (EMNLP-IJCNLP) [Internet]. Hong Kong, China: Association for Computational Linguistics; 2019 [cited 2026 Jan 13]. pp. 2567–77. 10.18653/v1/D19-1259

[28] Wu J, Zhu J, Qi Y, Chen J, Xu M, Menolascina F et al. Medical Graph RAG: Towards Safe Medical Large Language Model via Graph Retrieval-Augmented Generation [Internet]. arXiv; 2024 [cited 2026 Jan 13]. 10.48550/arXiv.2408.04187.

[29] Sohn J, Park Y, Yoon C, Park S, Hwang H, Sung M et al. Rationale-Guided Retrieval Augmented Generation for Medical Question Answering [Internet]. arXiv; 2025 [cited 2026 Jan 13]. 10.48550/arXiv.2411.00300.

[30] Jiang AQ, Sablayrolles A, Mensch A, Bamford C, Chaplot DS, Casas D, de las et al. Mistral 7B [Internet]. arXiv; 2023 [cited 2026 Jan 13]. 10.48550/arXiv.2310.06825

[31] Atri YK, Goyal V, Chakraborty T. Fusing Multimodal Signals on Hyper-complex Space for Extreme Abstractive Text Summarization (TL;DR) of Scientific Contents. Proceedings of the 29th ACM SIGKDD Conference on Knowledge Discovery and Data Mining [Internet]. Long Beach CA USA: ACM; 2023 [cited 2026 Jan 13]. pp. 3724–36. 10.1145/3580305.3599830

[32] Atri YK, Goyal V, Chakraborty T. Multi-Document Summarization Using Selective Attention Span and Reinforcement Learning. IEEE/ACM Trans Audio Speech Lang Process. 2023;31:3457–67. 10.1109/TASLP.2023.3316459.

[33] Atri Y, Iyer A, Chakraborty T, Goyal V. Promoting Topic Coherence and Inter-Document Consorts in Multi-Document Summarization via Simplicial Complex and Sheaf Graph. Proceedings of the. 2023 Conference on Empirical Methods in Natural Language Processing [Internet]. Singapore: Association for Computational Linguistics; 2023 [cited 2026 Jan 13]. pp. 2154–66. 10.18653/v1/2023.emnlp-main.133

[34] Dey A, Chowdhury T, Kumar Y, Chakraborty T. Corpora Evaluation and System Bias Detection in Multi-document Summarization. Online: Association for Computational Linguistics; 2020. pp. 2830–40. [cited 2026 Jan 13]. 10.18653/v1/2020.findings-emnlp.254. Findings of the Association for Computational Linguistics: EMNLP 2020 [Internet].

[35] Atri YK, Pramanick S, Goyal V, Chakraborty T. See, hear, read: Leveraging multimodality with guided attention for abstractive text summarization. Knowl Based Syst. 2021;227:107152. 10.1016/j.knosys.2021.107152.

[36] Tamkin A, Brundage M, Clark J, Ganguli D. Understanding the Capabilities, Limitations, and Societal Impact of Large Language Models [Internet]. arXiv; 2021 [cited 2026 Jan 13]. 10.48550/arXiv.2102.02503

[37] Li J, Yuan Y, Zhang Z, Enhancing LLM. Factual Accuracy with RAG to Counter Hallucinations: A Case Study on Domain-Specific Queries in Private Knowledge-Bases [Internet]. arXiv; 2024 [cited 2026 Jan 13]. 10.48550/arXiv.2403.10446

[38] Cai T, Tan Z, Song X, Sun T, Jiang J, Xu Y et al. FoRAG: Factuality-optimized Retrieval Augmented Generation for Web-enhanced Long-form Question Answering. Proceedings of the 30th ACM SIGKDD Conference on Knowledge Discovery and Data Mining [Internet]. Barcelona Spain: ACM; 2024 [cited 2026 Jan 13]. pp. 199–210. 10.1145/3637528.3672065

[39] Kim Y, Wu J, Abdulle Y, Wu H, MedExQA. Medical Question Answering Benchmark with Multiple Explanations. Proceedings of the 23rd Workshop on Biomedical Natural Language Processing [Internet]. Bangkok, Thailand: Association for Computational Linguistics; 2024 [cited 2026 Jan 13]. pp. 167–81. 10.18653/v1/2024.bionlp-1.14

