# Supplementary material for: A temporally Anchored Retrieval-Augmented Generation Framework for Metabolic and Bariatric Surgery Patient Education: An IFSO Artificial Intelligence Task Force Multinational Validation Study
Source: Obes Surg. 2026 May 20;36(6):3188–98. doi: 10.1007/s11695-026-08746-7 (PMC13249742; doi:10.1007/s11695-026-08746-7)
Supplement: Supplementary file 1 — Supplementary Material 1 [file 11695_2026_8746_MOESM1_ESM.docx]

**SUPPLEMENTAL TABLES**

Supplemental Table 1. Distribution of bariatric surgery-related questions across high-level categories

| Question Category | Questions (n) | Percentage (%) |
| --- | --- | --- |
| Preparation & Logistics | 68 | 5.22 |
| Surgical & Medical Info | 263 | 20.20 |
| Risks & Complications | 221 | 16.97 |
| Recovery & Lifestyle | 296 | 22.73 |
| Nutrition & Diet | 102 | 7.83 |
| Mental & Emotional Health | 287 | 22.04 |
| Cost & Insurance | 65 | 4.99 |

Supplemental Table 2. Sample questions for bariatric surgery content domains

| Question Category | Sample Questions |
| --- | --- |
| Risks & Complications | Are vitamin D levels and bone turnover markers related to non-alcoholic fatty liver disease in severely obese patients? |
|  | Does older age limit postbariatric surgery cognitive benefits: a preliminary investigation? |
| Recovery & Lifestyle | Does clinical trial demonstrate exercise following bariatric surgery improves insulin sensitivity? |
|  | Are serum markers of bone turnover increased at six and 18 months after Roux-en-Y bariatric surgery: correlation with the reduction in leptin? |
| Preparation & Logistics | Does a Pre-Hospital Patient Education Program improve Outcomes of Bariatric Surgery? |
|  | Does perioperative care map improve compliance with best practices for the morbidly obese? |
| Surgical & Medical Info | Is laparoscopic gastric bypass superior to laparoscopic gastric banding for treatment of morbid obesity? |
|  | Is potentially life-threatening sleep apnea unrecognized without aggressive evaluation? |
| Cost & Insurance | Does Medicare and Medicaid status predict prolonged length of stay after bariatric surgery? |
|  | Is medication cost significantly reduced after Roux-en-Y gastric bypass in obese patients? |
| Mental & Emotional Health | Are patient expectations of bariatric surgery gender specific – a prospective, multicenter cohort study? |
|  | Is support group meeting attendance associated with better weight loss? |
| Nutrition & Diet | Does dehydroepiandrosterone-sulfate modify human fatty acid composition of different adipose tissue depots? |
|  | Does low 25-hydroxyvitamin D affect insulin sensitivity in obesity after bariatric surgery? |

Supplemental Table 3. Linear mixed-effects analysis of expert Likert ratings using zero-shot configuration as the reference condition.

| System Configuration | Coefficient (Δ vs zero-shot) | 95% CI | p-value |
| --- | --- | --- | --- |
| Standard RAG | +0.55 | 0.45-0.65 | <0.001 |
| Context-prompted | +1.60 | 1.50-1.70 | <0.001 |
| Fine-tuned | +1.37 | 1.27-1.47 | <0.001 |
| bRAGgen | +2.19 | 2.09-2.29 | <0.001 |

System configuration modeled as fixed effect, while question and rater were included as random intercepts to account for repeated measurements and evaluator variability. Coefficients represent the estimated change in expert rating relative to the zero-shot baseline.

Supplemental Table 4. Hermes-Llama3-8B performance by question category zero-shot versus bRAGgen

| Question Category | Zero-shot | bRAGgen | ΔScore |
| --- | --- | --- | --- |
| Risks & Complications | 2.61 | 4.45 | +1.84 |
| Recovery & Lifestyle | 2.82 | 4.48 | +1.66 |
| Preparation & Logistics | 2.78 | 3.90 | +1.12 |
| Surgical & Medical Info | 2.96 | 4.33 | +1.37 |
| Cost & Insurance | 2.74 | 3.84 | +1.10 |
| Mental & Emotional Health | 2.56 | 4.40 | +1.84 |
| Nutrition & Diet | 2.93 | 4.16 | +1.23 |

ΔScore represents change in median score from 5-point Likert scale, 1 = lowest performance to 5 = highest performance

**SUPPLEMENTAL METHODS**

*Semantic knowledge caching*

To ensure rapid access to high-quality, previously validated clinical evidence, our system maintains a semantic knowledge cache $C$. This cache stores a fixed-size collection of query–document pairs $D=\{(q_{i},d_{i}){\}}_{i=1}^{N}$, where each query embedding $q_{i}\in R^{768}$ is generated using a domain-adapted SentenceTransformer, and $d_{i}$ is the corresponding clinical document. Given an incoming query $q$, the cache retrieves the most relevant document $d_{j}$ by maximizing cosine similarity:

$$C(q)={arg max}_{d_{j}\in D}\frac{q\cdot q_{j}}{\parallel q\parallel\parallel q_{j}\parallel} subject to \frac{q\cdot q_{j}}{\parallel q\parallel\parallel q_{j}\parallel}\geq\tau_{c},$$

where $\tau_{c}=0.7$ is a similarity threshold that filters out marginally relevant matches.

The cache is dynamically updated with new query–document pairs that pass clinical constraint checks and exhibit high generation confidence. It employs an age- and usage-aware eviction policy to preserve the most valuable entries. Specifically, when the cache reaches capacity, the entry with the lowest eviction score $\psi(d_{k})$ is removed:

$$\psi(d_{k})=\alpha f_{u}(d_{k})+(1-\alpha)e^{-t/\beta},$$

where $f_{u}(d_{k})$ is the document’s access frequency, $t$ is the time since its last use, $\beta$ is a decay constant, and $\alpha=0.6$ balances recency and popularity. Implemented with Faiss for efficient similarity search, the cache operates under a strict size limit (e.g., 500 entries) to guarantee sub-second retrieval latency in time-sensitive clinical workflows.

*Multi-Source Evidence Retrieval and Graph-Based Fusion*

When the semantic cache fails to yield a sufficiently relevant document (i.e., similarity $<\tau_{c}$), the system activates the multi-source retrieval engine $R$ to gather up-to-date evidence from external sources. This engine queries PubMed using extracted clinical keywords and supplements the results with a curated web search over trusted medical domains. Each retrieved result is structured as a document record containing text, source type (e.g., cache, pubmed, web), and extracted keywords. To synthesize these heterogeneous sources into a coherent and clinically trustworthy context, we introduce the evidence fusion module $F$. Given a set of $N$ retrieved documents $\{d_{1},\ldots,d_{N}\}$, $F$ constructs a heterogeneous graph where nodes represent documents and edges encode four relation types: same source, high embedding similarity ($>0.75$), shared clinical keywords (overlap $\geq2$), and PubMed priority (assigning higher credibility to peer-reviewed literature). Document embeddings are obtained using the same SentenceTransformer model employed in the cache. A relation-aware attention mechanism, RelationAttention, processes this graph:

$$H^{new}=\sum_{r=1}^{4} tanh(\alpha_{r})\cdot{Attn}_{r}(H;A_{r})+H,$$

where $H\in R^{N\times768}$ is the initial embedding matrix, $A_{r}$ is the adjacency matrix for relation $r$, and $\alpha_{r}$ are learnable relation weights. The updated node representations $H^{new}$ are then scored by their cosine similarity to the graph’s mean representation, yielding salience scores:

$$s_{i}=cos\left( h_{i}^{new}, \frac{1}{N}\sum_{j} h_{j}^{new} \right).$$

The top-$k$ ($k=6$) documents are selected based on softmax-normalized scores and concatenated with explicit separators to form the fused context:

$$fused\_context=d_{i_{1}} \parallel“-” \parallel d_{i_{2}} \parallel\cdots\parallel d_{i_{k}},$$

where $\parallel$ denotes string concatenation. This fused context preserves source diversity while emphasizing high-salience, cross-validated evidence, ensuring concordance across potentially conflicting medical sources.

*Uncertainty-Aware Generation with Clinical Constraint Reranking*

The adaptive text generation module $G$ produces clinically appropriate responses by combining stochastic decoding with rigorous safety validation. This prevents responses that violate established bariatric surgery guidelines. First, the system formats a concise prompt using the fused context and the original clinical query. Generation is performed via a large language model adapted with low-rank updates (LoRA) for bariatric domain alignment. To quantify predictive uncertainty, we employ Monte Carlo (MC) dropout during inference to enable the system to defer to retrieval when confidence is insufficient for safe clinical guidance. The model generates $S=6$ stochastic samples under active dropout, and for each sample $s_{i}$, we compute its negative log-likelihood (NLL) given the prompt. The resulting distribution over samples yields two key metrics:

- **Entropy**: $H=-\sum_{i=1}^{S} p_{i}logp_{i}$, where $p_{i}\propto exp(-{NLL}_{i})$,
- **Safe score**: $\sigma=1-H/logS$, which normalizes entropy to $[0,1]$ (higher = more confident).

However, high confidence does not guarantee clinical safety. To enforce domain-specific guidelines, we introduce a constraint-aware reranking mechanism. A lightweight patient profiler parses the input query to extract BMI and comorbidities (e.g., diabetes, hypertension) using pattern matching. A rule-based validator then evaluates each generated sample against bariatric surgery protocols, flagging violations such as claiming eligibility for patients with BMI $<35$ and no comorbidities, overconfident recommendations in borderline cases, advising against post-operative vitamin monitoring or follow-up, and recommending contraindicated medications (e.g., NSAIDs immediately post-op).

Samples are re-scored using a constrained utility function:

$$Score(s_{i})=-{NLL}_{i}-\lambda\cdot v_{i},$$

where $v_{i}$ is the number of violations in sample $s_{i}$, and $\lambda=0.5$ balances fluency and safety. The highest-scoring, minimally violating (or violation-free) sample is selected as the final answer. The output is further sanitized to remove model artifacts, role prefixes, and excessive verbosity, ensuring concise, professional clinical communication.

*Temporal Stability-Aware Inference*

To enable long-term reliability in an evolving clinical landscape, our temporal adaptation module $L$ implements Temporal Fisher Anchoring with Mechanism Selectivity (TFAMS). Unlike methods that rely on periodic fine-tuning or static regularization, TFAMS operates entirely at inference time, using the Fisher Information Matrix not as a training penalty, but as a *temporal sensor of knowledge stability*. TFAMS constructs a *Fisher trajectory* by estimating diagonal Fisher scores at real-world clinical checkpoints—such as the release of new ASMBS guidelines, FDA advisories, or expert-validated high-confidence interactions logged during deployment. For each model parameter $\theta_{j}$, it computes a temporal stability score:

$$S_{j}=\frac{\mu_{j}}{\sigma_{j}^{2}+\epsilon}, where \mu_{j}=\frac{1}{K}\sum_{k=1}^{K} F_{j}(t_{k}), \sigma_{j}^{2}=\frac{1}{K}\sum_{k=1}^{K} (F_{j}(t_{k})-\mu_{j})^{2},$$

with $F_{j}(t_{k})$ denoting the Fisher importance of $\theta_{j}$ at time $t_{k}$. Parameters with high $S_{j}$ are deemed to encode *stable clinical mechanisms* (e.g., NSAID contraindication), while those with low $S_{j}$ reflect historically volatile knowledge (e.g., BMI eligibility thresholds). TFAMS operates entirely at inference time and does not modify model weights. The Fisher stability statistics used by TFAMS are computed offline during periodic calibration (e.g., upon release of updated clinical guidelines) and stored as time-indexed snapshots for reuse during inference. This is distinct from the one-time LoRA domain adaptation described in Methods, which involves gradient-based fine-tuning prior to deployment.

During inference, $L$ uses $S_{j}$ to modulate generation behavior:

- Queries involving high-stability mechanisms trigger stricter constraint checking and lower generation temperature, anchoring responses to invariant truths.
- Queries aligned with low-stability regions allow higher fluency and greater reliance on real-time retrieval, facilitating adaptation to new evidence.
- Uncertainty and violation signals from $G$ are used to identify high-value interactions for future Fisher estimation, creating a closed-loop between safety, confidence, and temporal stability.

Fisher scores are computed offline or episodically (i.e., when incorporating new clinical guidelines or after aggregating a batch of expert-validated interactions). These Fisher estimates are stored as time-indexed snapshots and reused during inference, avoiding repeated computation and keeping deployment overhead low. System behavior is monotonic with respect to the stability threshold τ. Lowering τ reduces the frequency of online updates, while increasing τ increases adaptation frequency without changing retrieval or generation behavior. Across a wide range of τ values (0.45–0.65), we observe stable performance trends. λ_ewc acts as a continuous regularization knob rather than a hard constraint; increasing λ_ewc smoothly limits parameter drift without disabling adaptation.

Rather than defining explicit temporal checkpoints, TFAMS uses three implicit temporal anchors that are already present in the system pipeline: (i) retrieved external evidence such as PubMed abstracts, which naturally reflect evolving clinical consensus; (ii) expert-validated interaction traces selected through constraint-aware reranking, which act as locally validated behavioral signals; and (iii) the frozen pretrained model prior, which encodes long-term medical knowledge. The interaction between these anchors determines whether a query is treated as stability-critical or adaptation-permissive, without requiring explicit supervision over temporal boundaries. This provides a principled mechanism for *temporally adaptive inference*: the system “learns” what must never change and what may evolve, without any gradient-based retraining. In settings where periodic model updates are feasible, TFAMS can further guide safe fine-tuning by restricting updates to the subspace of volatile parameters—thereby preventing catastrophic interference with core clinical knowledge.

*End-to-End Inference Pipeline*

The complete inference workflow orchestrates all components into a unified, real-time decision support process. Given a clinical query $q$, the system proceeds as follows:

1. **Cache Lookup**: Query the semantic cache $C$. If a match with similarity $\geq\tau_{c}$ is found, its document is included; otherwise, proceed to multi-source retrieval.
2. **Evidence Retrieval**: Invoke $R$ to fetch documents from PubMed and trusted web sources using extracted keywords.
3. **Graph Fusion**: Combine all retrieved documents (cache + external) into a heterogeneous graph and apply $F$ to produce a salience-ranked, fused context.
4. **Stability-Aware Prompt Modulation (TFAMS)**: Estimate the temporal stability of the query’s semantic content using the precomputed Fisher trajectory. If the query aligns with high-stability clinical mechanisms (e.g., contraindications), generation temperature is reduced and constraint thresholds tightened; if aligned with volatile knowledge (e.g., evolving eligibility criteria), greater reliance is placed on the retrieved context.
5. **Stochastic Generation**: Use $G$ to generate $S=6$ candidate answers via MC dropout, computing NLLs, entropy, and safe scores for each.
6. **Constraint Reranking**: Parse patient attributes from $q$, evaluate clinical violations in each candidate, and select the highest-scoring compliant answer.
7. **Output Sanitization**: Trim redundant headers, query echoes, and excessive verbosity; enforce a maximum length of 120 words for clinical brevity.
8. **Logging and Temporal Checkpointing**: Record uncertainty metrics, violations, latency, and stability context. High-confidence, constraint-compliant interactions are logged as potential anchors for future Fisher trajectory updates, enabling continual refinement of the stability profile without model retraining.

This pipeline executes in under 5 seconds on average, meeting the latency requirements of interactive clinical settings. Critically, every stage is designed to be auditable: the fused context, salience scores, violation logs, uncertainty metrics, and stability context are all preserved in structured output artifacts, enabling retrospective review by clinicians or quality assurance systems. By integrating retrieval, fusion, temporal stability awareness, uncertainty, safety, and adaptability into a single coherent flow, our system delivers not just answers, but justifiable, trustworthy, and temporally coherent clinical recommendations.

*bRAGgen inference pipeline*

Input:

Clinical query q

Language model M

Semantic cache C

Retrieval modules R

Precomputed Fisher stability statistics F

Output:

Final answer a

1: // Evidence retrieval

2: D_cache ← Retrieve from semantic cache C using q

3: if D_cache is insufficient then

4: D_ext ← Retrieve from external sources R (e.g., PubMed, web)

5: else

6: D_ext ← ∅

7: end if

8: D ← D_cache ∪ D_ext

9: // Graph-based evidence fusion

10: Construct document graph G over D

11: Compute salience scores using relation-aware attention

12: Select top-k documents and concatenate into fused context X

13: // Prompt construction

14: p ← Format prompt using fused context X and query q

15: // Uncertainty-aware generation

16: S ← ∅

17: for i = 1 to N do

18: a_i ← Generate sample from M using stochastic decoding on p

19: Compute NLL_i for a_i given p

20: Add (a_i, NLL_i) to S

21: end for

22: Compute predictive entropy H over {NLL_i}

23: // Constraint-aware reranking

24: Parse patient profile Π from q

25: for each candidate (a_i, NLL_i) in S do

26: v_i ← Count clinical rule violations for a_i given Π

27: score_i ← −NLL_i − λ · v_i

28: end for

29: a_best ← argmax(score_i)

30: // TFAMS-lite stability modulation

31: Estimate stability signal s from Fisher statistics F and query q

32: if s indicates high stability then

33: Apply conservative decoding and stricter constraints

34: else

35: Allow greater reliance on retrieved evidence

36: end if

37: // Output sanitization

38: a ← Clean and truncate a_best for clinical clarity

39: return a
